# Probiotic intervention in colorectal cancer: the cytotoxic and apoptotic potential of *Lactobacillus* cell-free supernatants

**DOI:** 10.55730/1300-0152.2971

**Published:** 2026-06-01

**Authors:** Bahar YILMAZ, Berna ERDAL

**Affiliations:** 1 Department of Tumor Biology and Immunology, Institute of Health Sciences, Tekirdağ Namık Kemal University, Tekirdağ, Turkiye; 2 Department of Medical Microbiology, Faculty of Medicine, Tekirdağ Namık Kemal University, Tekirdağ, Turkiye

**Keywords:** Probiotic, *Lactobacillus bulgaricus*, *Lactobacillus paracasei*, cell-free supernatant, anticancer

## Abstract

**Background/aim:**

Failures in the treatment of colorectal cancer (CRC) have increased scientific interest in the potential of the bioactive compounds contained in cell-free supernatants (CFSs) obtained from probiotic bacteria for cancer treatment. The aim in this study was to investigate the effects of CFSs obtained from *Lactobacillus delbrueckii* subsp. *bulgaricus* (*L. bulgaricus*) and *Lactobacillus paracasei* subsp. *paracasei* (*L. paracasei*) on colorectal cancer cell lines (HT-29 and Caco-2) and a healthy colon epithelial cell line (CCD841 CoN).

**Materials and methods:**

Cytotoxic effects on the cell lines were determined by MTT assay. In line with the IC_50_ values obtained, morphological changes were examined by fluorescent cell staining and cell cycle analyses were performed. In addition, the expression levels of the apoptosis-related genes Bax, Cas 9, Cas 8, Cas 3, and Bcl-2 in CFS-treated cells were measured using the qRT-PCR method.

**Results:**

The MTT assay revealed that CFSs exerted a significant cytotoxic effect on cancer cells. Assessments with AO/PI fluorescent staining confirmed the increase in the number of apoptotic cells. Cell cycle analyses showed a tendency for accumulation in the G0/G1 phase and a decrease in the S phase. Furthermore, in cells treated with inactive CFS, proapoptotic genes (Bax, Cas 9, Cas 8, and Cas 3) were significantly increased, while antiapoptotic Bcl-2 gene expression decreased.

**Conclusion:**

This study demonstrates that both live and inactivated *Lactobacillus* CFSs can exert targeted anticancer effects in Caco-2 and HT-29 cells. These results indicate that CFSs may be a potential biotherapeutic option for cancer treatment in the future.

## 1. Introduction

Colorectal cancer (CRC) is among the most common malignancies in both men and women worldwide and continues to play a significant role in cancer-related deaths (Rawla et al., 2019). The incidence of CRC is largely related to lifestyle, environmental factors, and dietary habits (Siegel et al., 2018). Different treatment strategies such as surgery, chemotherapy, radiotherapy, and immunotherapy are applied to manage the disease (Fillon, 2022).

Studies examining the relationship between gastrointestinal microbiota and CRC have been increasing in recent years (Lazar et al., 2018; Gomaa, 2020). Probiotic bacteria, which are important members of the microbiota, are thought to have the potential to regulate inflammation, modulate immune responses, and affect cancer development (Garrett, 2015; Yoo et al., 2020). These bacteria help maintain epithelial barrier integrity by producing various metabolites in the intestinal environment and can suppress inflammatory processes (Sanders et al., 2018).

Cell-free supernatants (CFSs), which are postbiotic components released during bacterial growth, contain active substances such as antimicrobial peptides, organic acids, and hydrophobic compounds. These components have been reported to exhibit antimicrobial, antiinflammatory, and immunomodulatory effects (Gomaa, 2020; Kim and Lee, 2022; Khaleel et al., 2023). *Lactobacillus* species, known for their probiotic properties, are particularly prominent in therapeutic studies (Eslami et al., 2019; Molska and Reguła, 2019; Tripathy et al., 2021). Especially *Lactobacillus delbrueckii* subsp. *bulgaricus* (*L. bulgaricus*) and *Lactobacillus paracasei* subsp. *paracasei* (*L. paracasei*) strains were selected for investigation in the present study due to their well-defined probiotic properties, bioactive metabolite production capacity, and previously reported immunomodulatory and antiproliferative effects. These strains are commonly used in fermented foods and have been associated with regulating gut microbiota composition, which is closely linked to colorectal cancer pathogenesis (Karimi Ardestani et al., 2019; Aguilar et al., 2025). Inactive derivatives and metabolic byproducts of *Lactobacillus* strains have attracted increasing attention in recent years due to their reported antiproliferative and apoptotic effects (Karimi Ardestani et al., 2019; Kim et al., 2022). Recent studies have shown that the biological effects of probiotics can vary depending on strain-specific characteristics, viability status, and the host (Nowak et al., 2019; Markowiak-Kopeć and Śliżewska, 2020; Liu et al., 2023; Shah et al., 2024; Aguilar et al., 2025). The aim of the present study was to investigate the antiproliferative and apoptotic effects of CFSs from *L. bulgaricus* and *L. paracasei* on the human colorectal adenocarcinoma cell lines Caco-2 and HT-29.

## 2. Materials and methods

### 2.1. Ethical approval

The study was approved by the Ethics Committee of the Faculty of Medicine, Tekirdağ Namık Kemal University (approval no: 2021.289.12.12; December 28, 2021), and all experimental procedures were conducted in accordance with the Declaration of Helsinki. Since the study involved only in vitro (cell line) models, written informed consent from human participants was not required.

### 2.2. Preparation of probiotics and their CFSs

In the present study, *L. bulgaricus* and *L. paracasei* isolates amplified by the 16S rDNA gene region PCR method and sequenced by Tokatlı Demirok et al. (2023) were used. The isolates were inoculated onto de Man, Rogosa, and Sharpe (MRS) agar (401728S2, Biolife, Milan, Italy) and incubated under anaerobic conditions at 37 °C for 24–48 h. Following this step, the cultures were inoculated into MRS liquid medium (4017292, Biolife) and inactivated at 100 °C for 30 min in a water bath (de Man et al., 1960). After inactivation, the cultures were centrifuged at 4500 rpm for 20 min to obtain the CFSs. These CFSs were sterilized using 0.22-μm PES filters (1.094.07.006.050, Isolab, Eschau, Germany) and the efficacy of inactivation was verified on MRS agar (Sadeghi-Aliabadi et al., 2014; Karimi Ardestani et al., 2019). In line with preliminary studies and the literature reporting that the anticancer effects of probiotic-derived CFS are evaluated based on percentage (v/v) concentrations, CFS samples were administered as two-fold serial dilutions in the range of 0.39%–100% (Hoxha et al., 2023; Erdal et al., 2025).

### 2.3. Cell culture

HT-29 and Caco-2 cell lines were chosen because they are well-characterized in vitro models of colorectal cancer and are commonly used to evaluate the antiproliferative effects of probiotic-derived metabolites. The CCD 841 CoN cell line was included to assess the selectivity of the observed cytotoxic effect against healthy colon epithelium. The HT-29 (ATCC HTB-38) and Caco-2 (ATCC HTB-37) cancer cell lines and CCD 841 CoN (ATCC CRL-1790) healthy colon epithelial cell line, 10% fetal bovine serum (FBS, P30-3306, Pan Biotech, Aidenbach, Germany), 2 mM L-glutamine (25030-024, Gibco, Paisley, UK), and 1% penicillin/streptomycin (15140-148, Gibco, New York, NY, USA) in Dulbecco’s modified Eagle’s medium (ECM0103L, Euroclone, Milan, Italy), in a 37 °C incubator with a 5% CO_2_ and 95% humidity atmosphere.

### 2.4. Cytotoxicity assays

Cells that reached 80%–90% confluence in the MTT assay were passaged and seeded into 96-well plates at 100 μL per well to achieve a density of 1 × 10^4^ cells/well. After 24 h of incubation, live and inactive CFS samples at concentrations ranging from 0.39% to 100% were applied. To evaluate the early cytotoxic responses caused by probiotic-induced CFS, the samples were incubated for 24 h. MTT solution (475989-1GM, Merck, Darmstadt, Germany) (5 mg/mL) was added to each well at 10 μL and, after 4 h of incubation, the formazan crystals formed were dissolved in 100 μL of dimethyl sulfoxide (1264ML100, Neofroxx, Einhausen, Germany), and the absorbances were measured at 570 nm (BioTek, Winooski, VT, USA). Cell viability percentage was calculated using the formula ([OD]Test − [OD]Blank)/([OD]Control − [OD]Blank) × 100% (Yıkmış et al., 2024; Zhao et al., 2024). All experiments were performed in triplicate and three independent biological replicates. IC_50_ values were analyzed using GraphPad Prism 8.0.

### 2.5. Acridine orange/propidium iodide staining

The cellular effects of live and inactive CFS at IC_50_ concentrations in HT-29, Caco-2, and CCD841 CoN cells were evaluated using the AO (A6014-10G, Sigma, St. Louis, MO, USA)/PI (P4170-10MG, Sigma) dual fluorescent staining method. After adding 1 × 10^4^ cells to each well, the IC_50_ bacterial CFSs were added (Erdal et al., 2025). Then 20 μL of dye solution was added to each well, the wells were incubated for 5 min, and images were captured using a fluorescence microscope and analyzed with the software Cytovision Capture Station (v7.5; Genetix; Leica Microsystems, Wetzlar, Germany). Fluorescence detection was performed using filter sets appropriate for AO and PI staining; AO fluorescence was detected at approximately 480 nm excitation and 520 nm emission, while PI fluorescence was detected at approximately 520 nm excitation and 620 nm emission (Wunsch et al., 2015). All experiments were performed in triplicate and three independent biological replicates.

### 2.6. Cell cycle assay

Cells were seeded at a density of 2.5 × 10^5^ cells/well in six-well plates. Cells were treated with live or inactivated CFS at IC_50_ concentrations following incubation. After treatment, the cells were fixed with 70% ethanol and kept at +4 °C overnight. After fixation, a DPBS solution containing 0.1% Triton X-100 and 3.3 μL of RNase was applied, and the sample was incubated at 37 °C for 30 min. The cells were then stained with 10 μL of PI and incubated in the dark for 1 h. All experiments were performed in triplicate and three independent biological replicates. The cell suspensions prepared were analyzed using a DxFLEX B4-R2-V3 Flow Cytometer Autoloader (Beckman Coulter, Amersham, UK) to calculate the distribution in the Sub-G, G0/G1, S, and G2/M phases (Chen et al., 2023).

### 2.7. Evaluation of mRNA expression by real-time PCR

Following the application of live and inactivated CFS at IC_50_ doses to the cells, changes in the expression levels of apoptosis-related genes were investigated. For this purpose, total RNA was isolated from the cells using a PureLink RNA Mini Kit (Thermo Fisher Scientific, Carlsbad, CA, USA). The purity and concentration of the RNA isolated were determined using a NanoDrop spectrophotometer (AllSheng, Hangzhou, China). cDNA was synthesized from the RNA samples obtained using a High-Capacity cDNA Reverse Transcription Kit (ThermoFisher, Vilnius, Lithuania). Gene expression analyses were performed using the qPCR method on a LightCycler 2.0 (Roche, Basel, Switzerland) device with SYBR Green-based FastStart Essential DNA Green Master Mix (Roche). In the present study, the expression of apoptosis-related genes, including BAX, Cas 3, Cas 8, Cas 9, and Bcl-2, was evaluated, with GAPDH as the reference gene. Gene expression changes were analyzed using the ΔΔCt method based on Ct values and calculated using the 2^−ΔΔCt^ formula (Livak and Schmittgen, 2001). All experiments were performed in triplicate and three independent biological replicates. The primer sequences used in the study and their corresponding amplicon lengths are presented in Table.

### 2.8. Statistical analysis

All experimental data are presented as the mean ± standard deviation (SD) of three replicates. Excel (Version 365, Microsoft Corporation, Redmond, WA, USA) and GraphPad Prism (Version 8.0, GraphPad Software Inc., San Diego, CA, USA) were used to prepare the graphs. The data were analyzed using ANOVA, while Tukey’s post-hoc test was applied for multiple comparisons. Statistically significant results (p ≤ 0.05) are indicated in the relevant graphs.

## 3. Results

### 3.1. Cytotoxicity results

**Figure 1:**
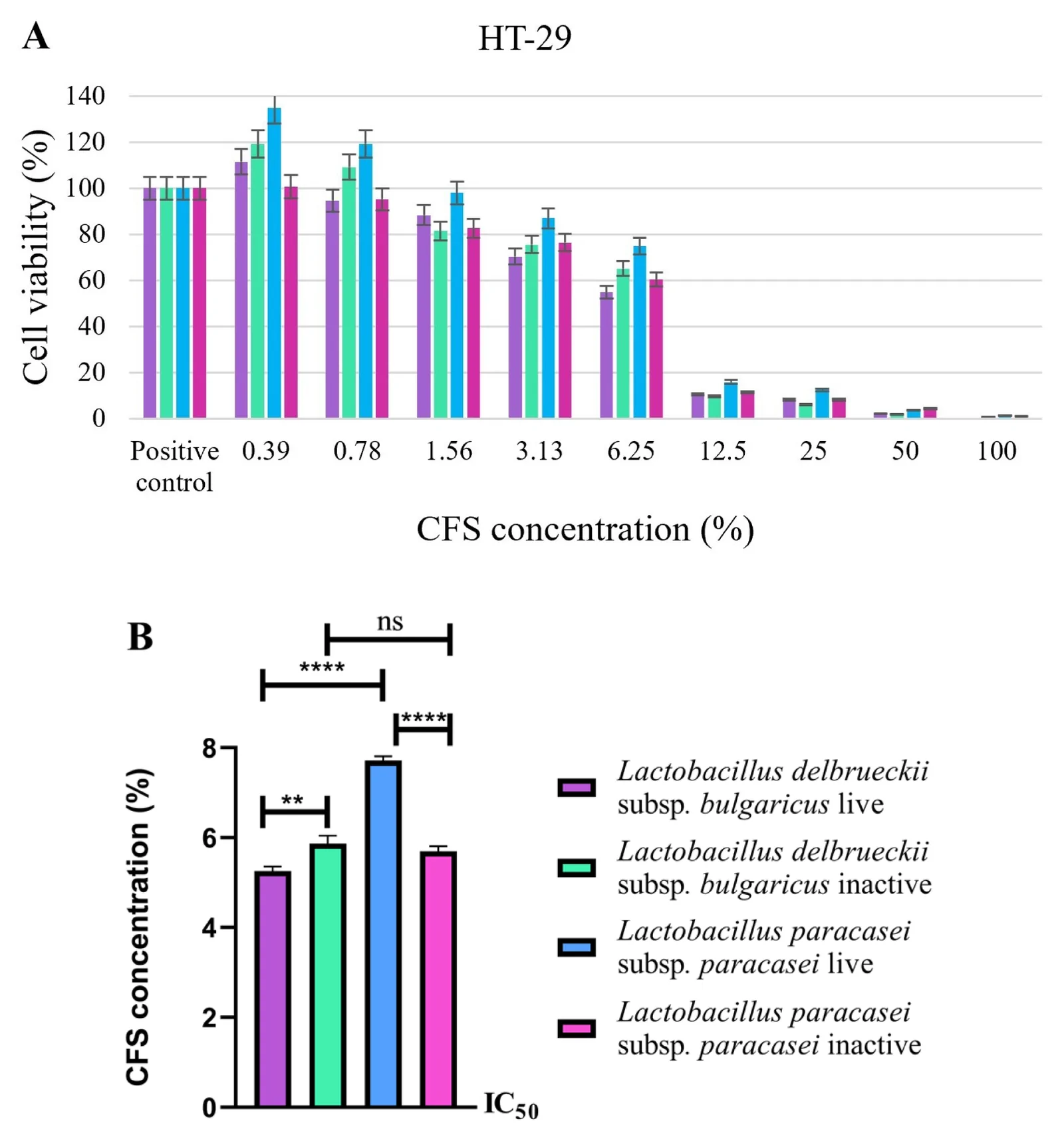
MTT (A) and IC_50_ (B) results for *L. bulgaricus* and *L. paracasei* CFS in HT-29 cells (**p *<* 0.0014, ****p *<* 0.0001, ns: not significant).

Both live and inactivated CFS isolates of *L. bulgaricus* and *L. paracasei* caused a significant loss of cell viability in the HT-29 cell line in a concentration-dependent manner (Figure 1). The cytotoxic effect observed compared to the positive control group was statistically significant (p < 0.0001). This indicates that both *Lactobacillus* CFSs have the potential to inhibit HT-29 cell proliferation in a dose-dependent manner. The IC_50_ values for *L. bulgaricus* were 5.255 ± 0.102% for live CFS and 5.872 ± 0.171% for inactive CFS. For *L. paracasei*, the IC_50_ values were 7.714 ± 0.099% for live CFS and 5.695 ± 0.114% for inactive CFS (Figure 1). CFSs from both *Lactobacillus* species exhibited cell-proliferation-inhibitory effects in HT-29 cells. These effects were observed in both live and inactivated CFSs, with differences in IC_50_ values suggesting differences in cytotoxic potential between species and form types. In particular, the lower IC_50_ value of *L. bulgaricus* live CFS indicates a potent antiproliferative effect, while the lower IC_50_ value of *L. paracasei* inactivated CFS suggests that heat-stable bioactive compounds may be present in this supernatant.

**Figure 2:**
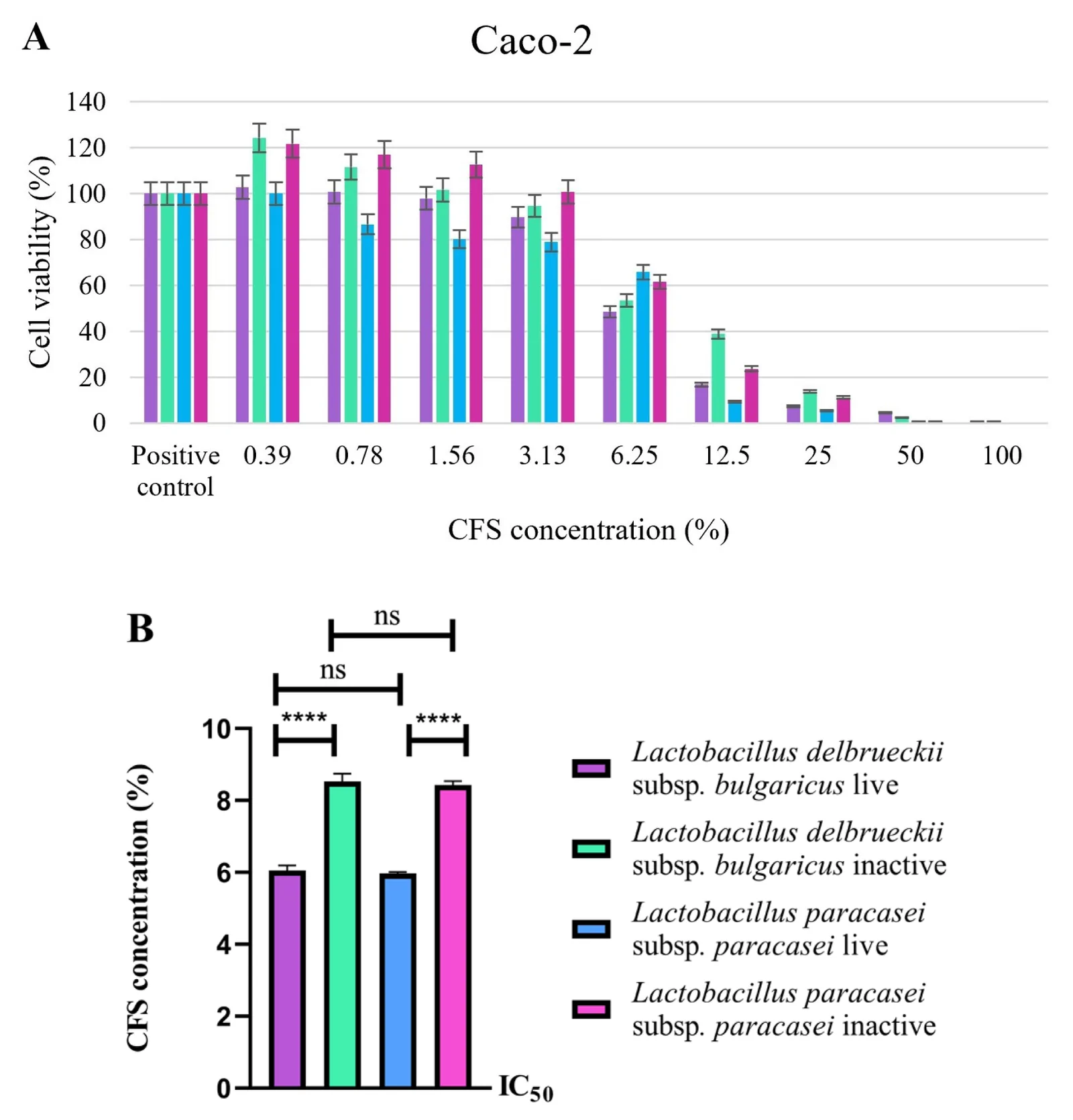
MTT (A) and IC_50_ (B) results for *L. bulgaricus* and *L. paracasei* CFS in Caco-2 cells (****p *<* 0.0001, ns: not significant).

Similar to the findings observed in HT-29 cells, a dose-dependent decrease in cell viability was detected after 24-h incubation with live and inactive CFS from *L. bulgaricus* and *L. paracasei* isolates in the Caco-2 cell line (Figure 2). Especially at high CFS concentrations, the CFS of both *Lactobacillus* species was found to significantly suppress cell viability compared to the positive control group (p < 0.0001). The IC_50_ values for *L. bulgaricus* were 6.057 ± 0.138% for viable CFS and 8.530 ± 0.216% for inactive CFS. For *L. paracasei*, the IC_50_ values were 5.970 ± 0.039% for live CFS and 8.430 ± 0.109% for inactive CFS (Figure 2). These findings indicate that both *Lactobacillus* CFSs exhibit antiproliferative effects on Caco-2 cells. The IC_50_ values of live CFSs from *L. bulgaricus* and *L. paracasei* were quite close to each other, while the IC_50_ values of inactive CFSs were higher than those of live CFSs.

**Figure 3:**
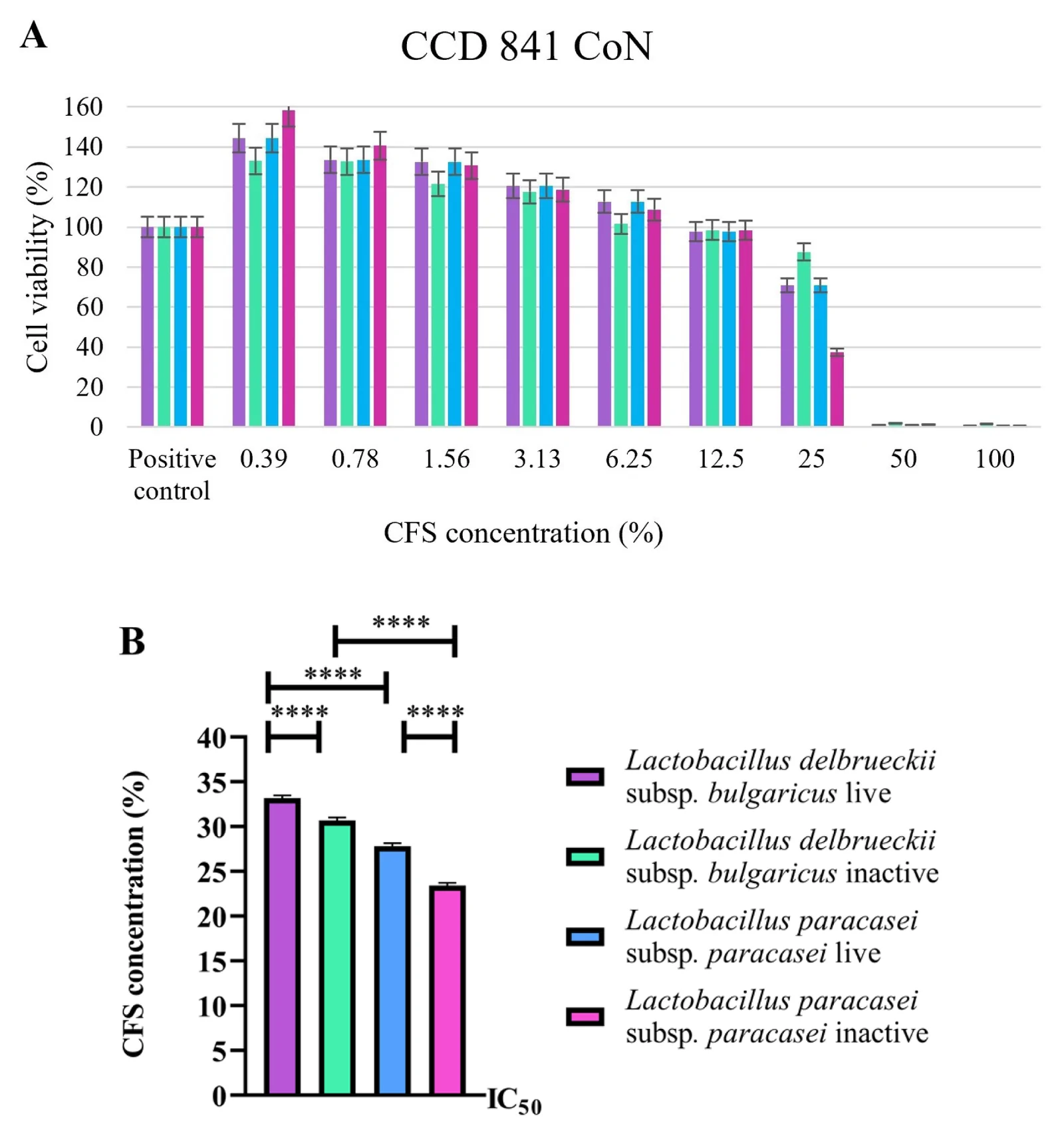
MTT (A) and IC_50_ (B) results for *L. bulgaricus* and *L. paracasei* CFS in CCD 841 CoN cells (****p *<* 0.0001).

It was observed that live and inactive CFS of *L. bulgaricus* and *L. paracasei* isolates reduced the viability of CCD 841 CoN cells at high concentrations (Figure 3). Consistent with results in cancer cell lines, a statistically significant decrease in CCD 841 CoN viability was observed with increasing CFS concentration (p < 0.0001). The IC_50_ values were 33.196 ± 0.272% (live) and 30.682 ± 0.324% (inactive) for *L. bulgaricus*; for *L. paracasei*, they were 27.802 ± 0.342% (live) and 23.400 ± 0.303% (inactive) (Figure 3). These IC_50_ values were higher than those determined in HT-29 and Caco-2 cell lines.

### 3.2. Fluorescent staining results

**Figure 4:**
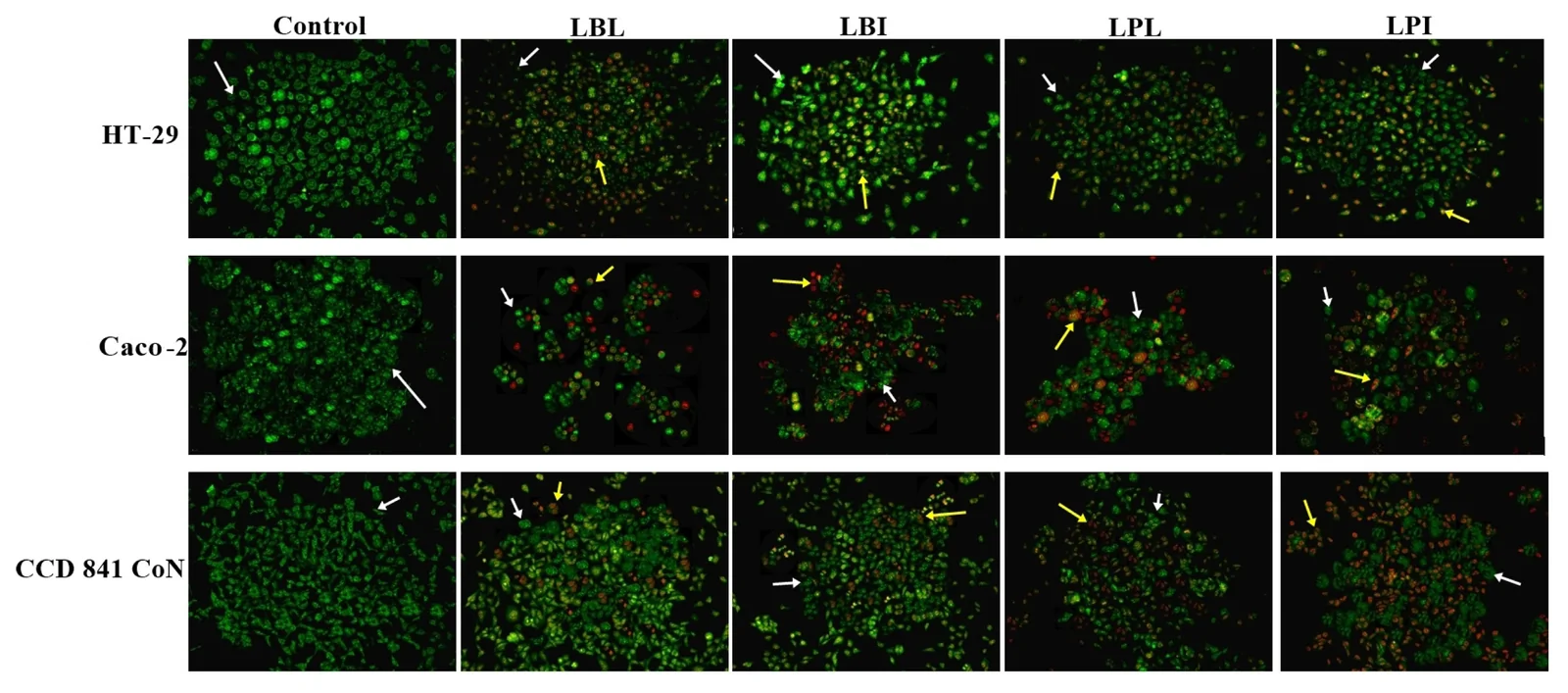
Fluorescence microscope image (20×) of live and inactive CFSs applied at IC_50_ concentrations in HT-29, Caco-2, and CCD 841 CoN cells (white arrow: live cells, yellow arrow: apoptotic cells; LBL: *L. bulgaricus* live, LBI: L. *bulgaricus* inactive, LPL: *L. paracasei* live, LPI: *L. paracasei* inactive).

**Figure 5:**
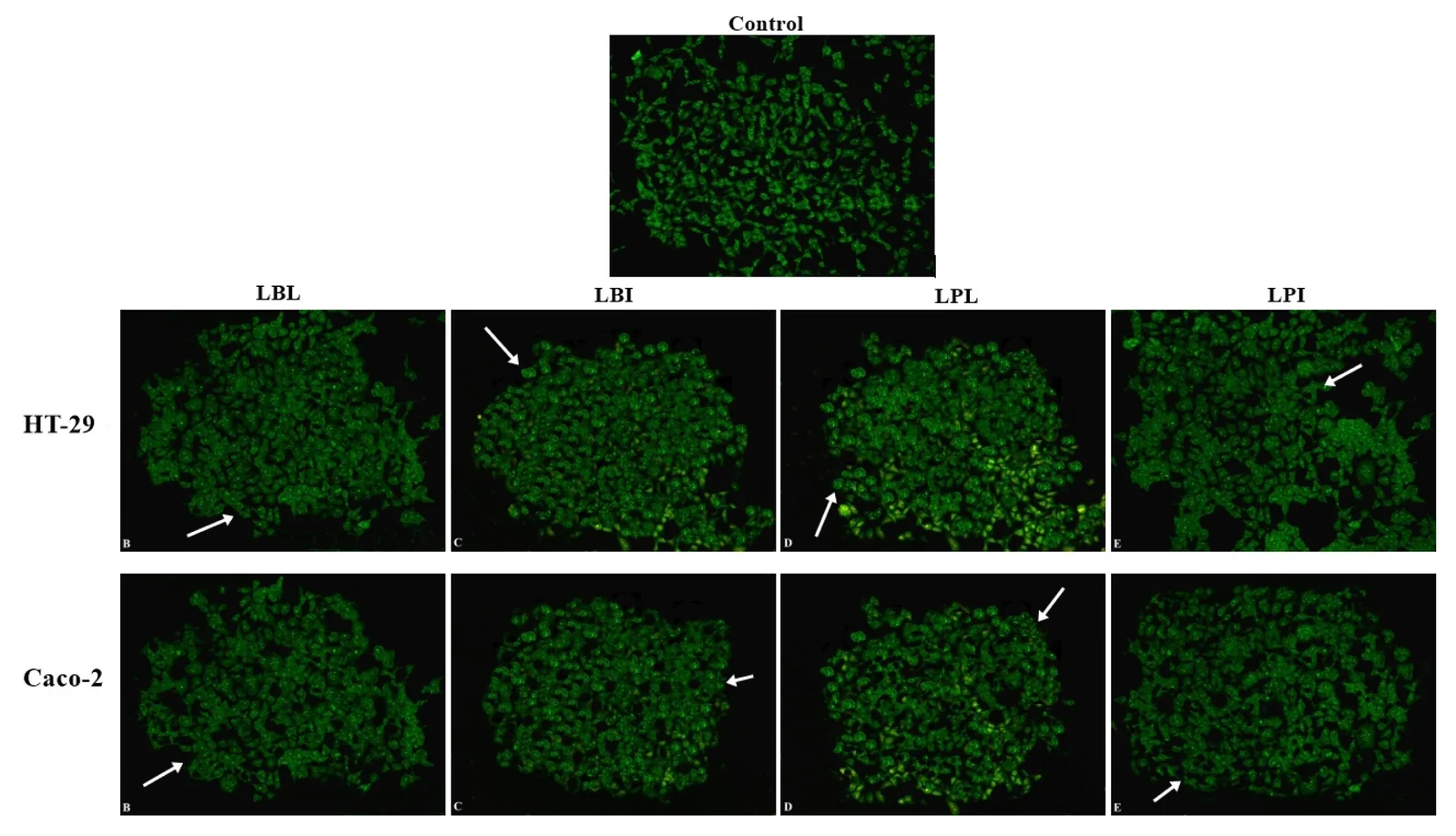
Fluorescence microscope image (20×) obtained after applying live and inactive CFSs to CCD841-CoN cells at IC_50_ concentrations determined for cancer cells (white arrow: live cells; LBL: *L. bulgaricus* live, LBI: *L*. *bulgaricus* inactive, LPL: *L. paracasei* live, LPI: *L. paracasei* inactive).

AO/PI fluorescence staining analyses revealed that probiotic CFS administration exhibited a dose-dependent cytotoxic effect in colorectal cancer cell lines and induced apoptosis (Figure 4). To evaluate the selective cytotoxic effect of these CFSs, the effects of the IC_50_ concentrations determined for colorectal cancer cell lines on CCD841 CoN cells were also assessed. AO/PI analyses performed on healthy cells at these concentrations showed that no significant apoptotic or cytotoxic morphological changes observed in cancer cells occurred (Figure 5).

### 3.3. Cell cycle results

**Figure 6:**
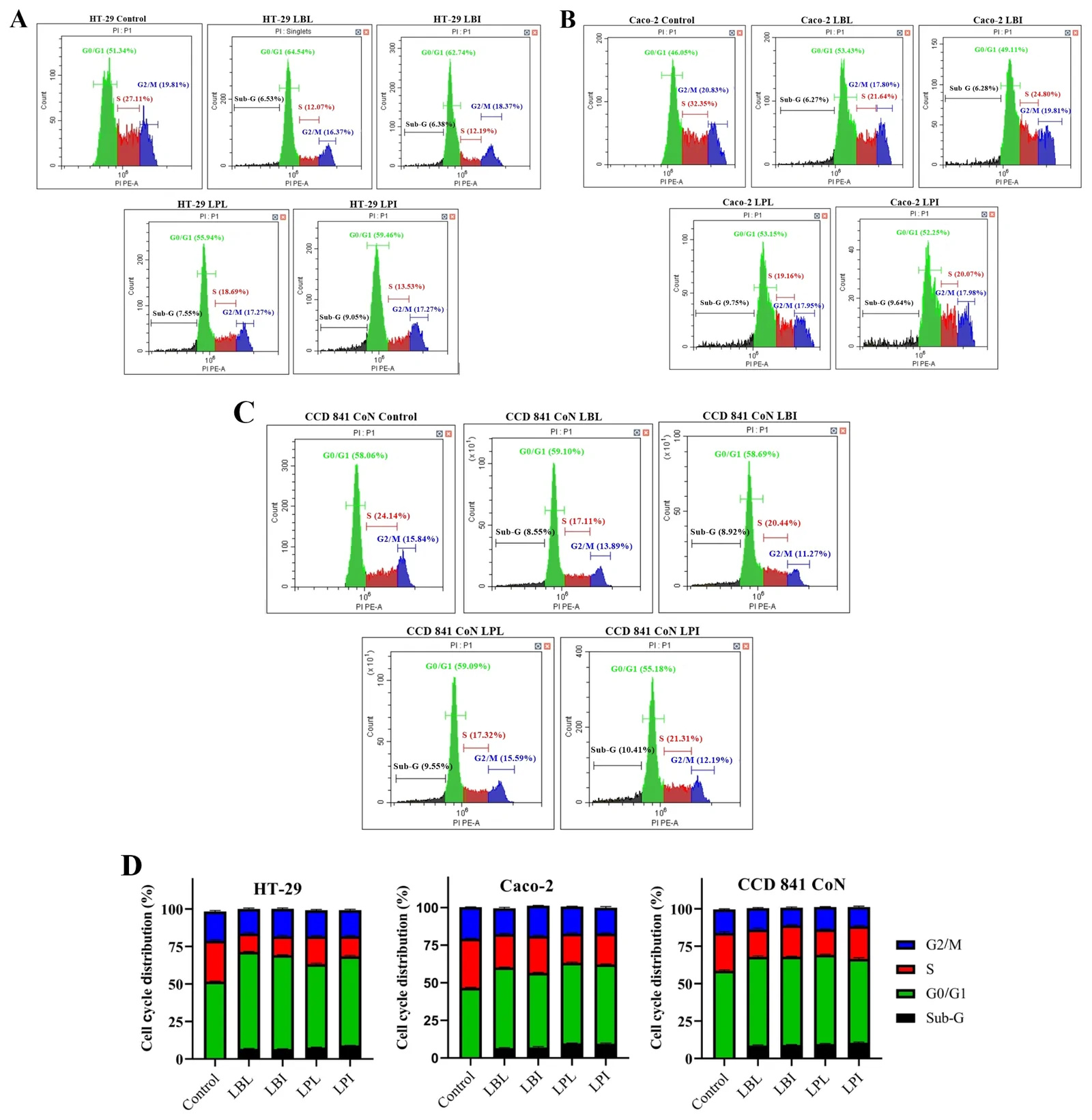
Effect of IC_50_ application of live and inactivated CFSs on the HT-29 (A), Caco-2 (B), and CCD841-CoN (C) cell cycle. Flow cytometry analysis images (D) Percentage distribution of cell cycle phases (LBL: *L*. *bulgaricus* live, LBI: *L*. *bulgaricus* inactive, LPL: *L*. *paracasei* live, LPI: *L*. *paracasei* inactive).

It was determined that probiotic bacterial CFSs caused arrest at different rates in the cell cycle phases of the tested cells (Figure 6). Compared with the control group, when the IC_50_ concentrations of *L. bulgaricus* and *L. paracasei* CFS were applied to HT-29 cells, the percentage changes in the G0/G1 phase were significant (p < 0.05). *L. bulgaricus* live CFS caused a 13.02% increase in G0/G1 phase arrest, while inactive CFS caused an 11.1% increase; *L. paracasei* live CFS caused a 3.97% increase in G0/G1 phase arrest, while inactive CFS caused a 7.77% increase. In Caco-2 cell analyses, CFS from *L. bulgaricus* isolates caused cell accumulation in the G0/G1 phase at rates of 7.01% (live) and 2.99% (inactive). In comparison, CFS from *L. paracasei* isolates caused cell accumulation at rates of 7.04% (live) and 6.02% (inactive), resulting in phase arrest. In CCD841 CoN cells, CFS also induced cell cycle accumulation. Compared to the control group, live *L. bulgaricus* CFSs caused a 0.7% increase and inactive CFSs caused a 0.31% increase in the G0/G1 phase; live *L. paracasei* CFSs caused a 0.93% increase and inactive CFSs caused a 1.3% increase in accumulation. Additionally, the Sub-G cell ratio was higher in all studied cells, particularly in *L. paracasei* inactive CFS, compared with the control group. The decreased live cell density in the S and G2/M phases indicates that the cells remained in the G0/G1 phase. These findings support the inhibition of cell proliferation.

### 3.4. Gene expression analysis results

**Figure 7:**
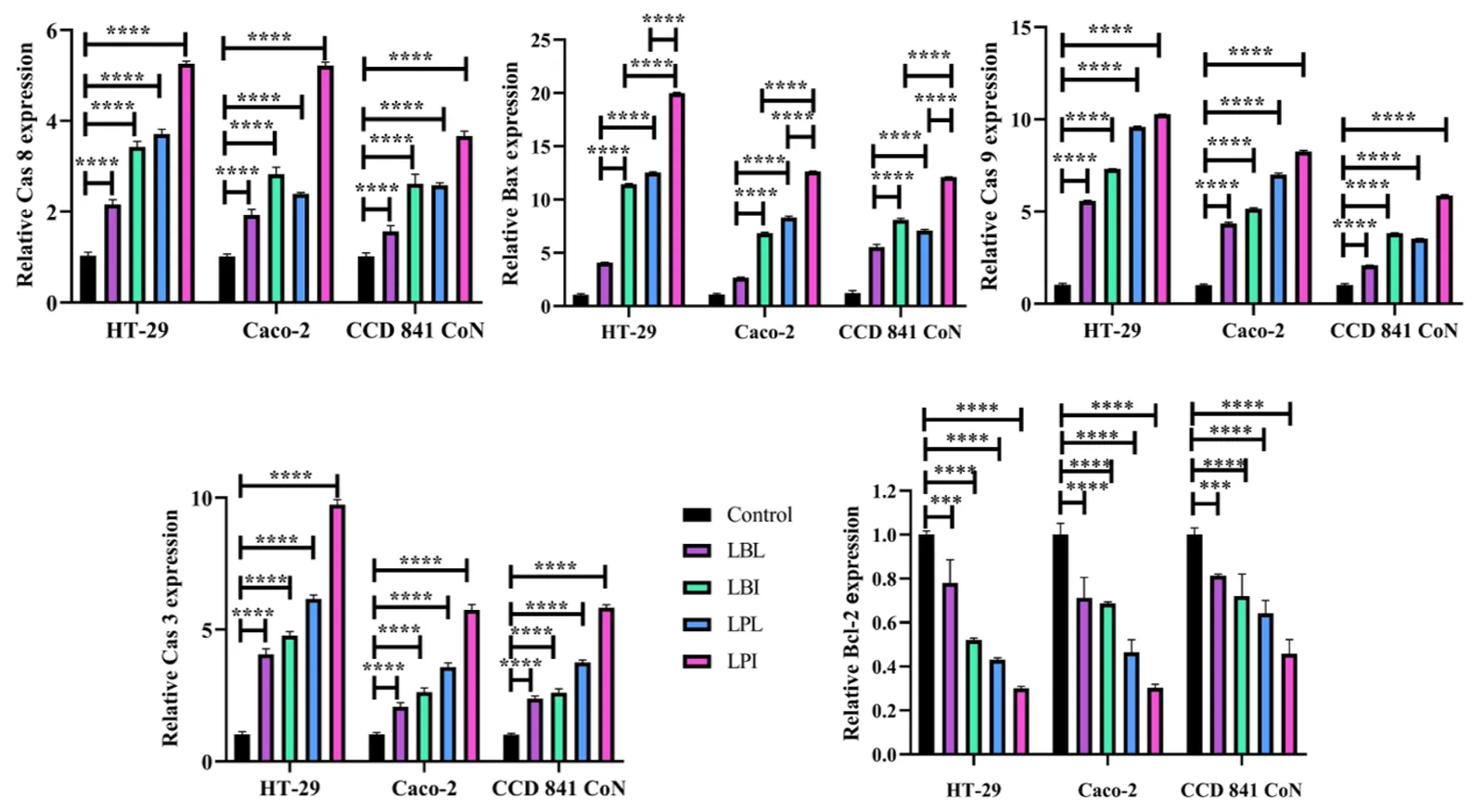
Changes in gene expression in HT-29, Caco-2, and CCD 841 CoN cells following IC_50_ treatment with CFSs (LBL: *L. bulgaricus* live, LBI: *L. bulgaricus* inactive, LPL: *L. paracasei* live, LPI: *L. paracasei* inactive, ***p = 0.0009, ****p < 0.0001).

The CFSs applied were observed to cause significant increases in the expression of the Bax, Cas9, Cas8, and Cas3 genes in HT-29, Caco-2, and CCD 841 CoN cells (p < 0.0001; Figure 7). In the analyses of Bax gene expression, it was observed that the inactive forms of *L. bulgaricus* and *L. paracasei* caused a greater increase in gene expression than their live forms. In HT-29 cells, the inactive CFS of *L. bulgaricus* increased Bax expression by 11.45 ± 0.06-fold compared to the control group, while the inactive form of *L. paracasei* caused an increase of 19.99 ± 0.05-fold. Similarly, in Caco-2 and CCD841-CoN cells, inactive forms were also more effective at increasing Bax expression (p < 0.0001).

**Figure 8:**
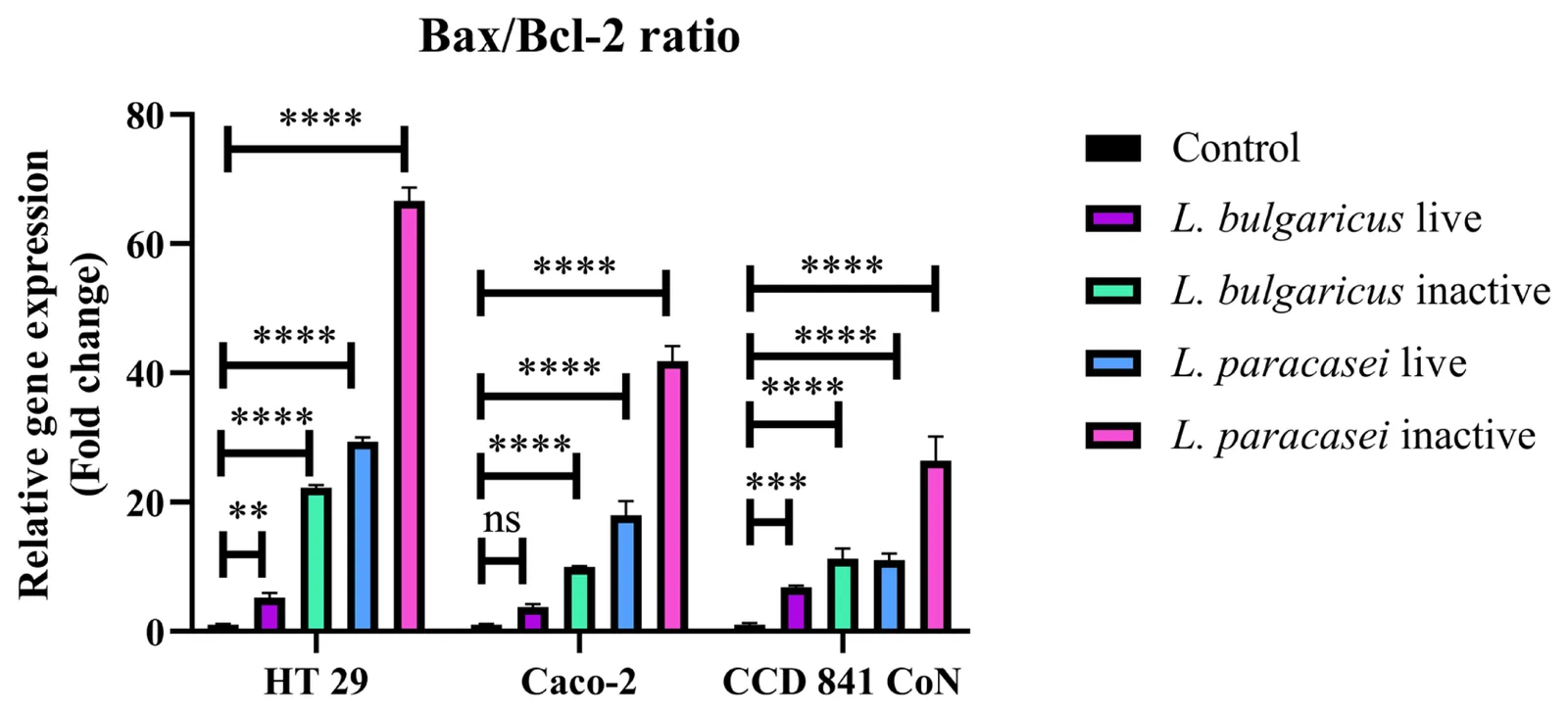
Changes in the BAX/Bcl-2 ratio in HT-29, Caco-2, and CCD 841 CoN cells following IC_50_ treatment with CFSs (***p = 0.0002; ****p < 0.0001).

The expression of the Cas9, Cas8, and Cas3 genes increased significantly in all cells compared to the control group. However, inactive CFSs had more potent effects on gene expression. In HT-29 cells, inactive CFSs from *L. bulgaricus* increased Cas 9 gene expression by 7.32 ± 0.01-fold, Cas 8 gene expression by 3.43 ± 0.12-fold, and Cas 3 gene expression by 4.77 ± 0.15-fold; while the inactive CFS of *L. paracasei* caused a 10.28 ± 0.01-fold increase in Cas9, a 5.26 ± 0.05-fold increase in Cas8, and a 9.74 ± 0.19-fold increase in Cas3. It was determined that inactive CFSs tend to suppress Bcl-2 expression across all cells studied, and that this gene exhibits an antiapoptotic effect, unlike other genes. In HT-29 cells, the inactive CFS of *L. bulgaricus* reduced Bcl-2 expression to 0.52 ± 0.01-fold, while the inactive CFS of *L. paracasei* caused a more pronounced decrease to 0.30 ± 0.01-fold. A similar significant reduction in BCL-2 expression was observed in Caco-2 and CCD841-CoN cells (p < 0.0001). The Bax/Bcl-2 gene expression ratios are shown in Figure 8. The results showed that the Bax/Bcl-2 ratio increased significantly compared with the control group, particularly following treatment with inactive CFS of *L. paracasei* in the HT-29 and Caco-2 cell lines. In contrast, the changes in the Bax/Bcl-2 ratio in CCD 841 CoN were more limited (p < 0.0001).

## 4. Discussion

In recent years, the potential of probiotic bacteria and the bioactive compounds they produce in cancer treatment has attracted attention (Ranjbar et al., 2019; Thoda and Touraki, 2023; Gholizadeh et al., 2024). Specifically, CFSs derived from *Lactobacillus* species have been reported to be effective in reducing cell viability, arresting the cell cycle, and increasing oxidative stress in colorectal cancer cells (Sivamaruthi et al., 2020; O’Flaherty et al., 2023). Consistent with these observations, in the present study the antiproliferative and apoptotic effects of CFSs derived from *L. bulgaricus* and *L. paracasei* on HT-29 and Caco-2 cells were comprehensively demonstrated. These effects were evidenced by a significant decrease in cell viability, cell cycle arrest, morphological apoptotic changes confirmed by AO/PI staining, and modulation of apoptotic gene expression.

The results of some studies using cytotoxicity and fluorescence methods are consistent with our study’s findings. Erfanian et al. (2023) reported that CFS derived from *Lactobacillus acidophilus* inhibited the proliferation of HT-29 cells in a dose- and time-dependent manner, while showing low toxicity in healthy human dermal fibroblast cells. Similarly, it has been shown that CFSs obtained from different lactic acid bacteria strains exhibit significant antiproliferative effects in Caco-2, cervical cancer (HeLa), and Madin–Darby bovine kidney cells, and this effect increases with increasing dose (Nowak et al., 2022; Hoxha et al., 2023). Recent studies have shown that CFSs, particularly those derived from *Lactobacillus plantarum* and *Lactobacillus brevis* strains, exhibit potent anticancer effects and induce apoptosis. Salek et al. (2024) reported that CFSs, when combined with the chemotherapeutic agent 5-fluorouracil, reduced the viability of HT-29 and healthy embryonic kidney (HEK-293) cells. These results suggest that CFSs may have promising potential in combination therapies (Salek et al., 2024). Similarly, Chuah et al. (2019) demonstrated that *L. plantarum* CFSs induced significant cytotoxic effects in different cancer cell lines, including MCF-7, HeLa, HT-29, HEP-G2, K562, and HL60. All these studies demonstrate that *Lactobacillus*-derived CFSs exhibit cytotoxic effects on cancer cells. The high IC_50_ values observed in CCD841 CoN cells in the present study indicate that these CFSs possess selective cytotoxic potential on healthy cells and are safe to apply. However, the marked reduction in cell viability observed between 25% and 50% CFS concentrations in CCD841 CoN cells suggests that the cytotoxic response may not increase linearly with concentration. This phenomenon could be related to the concentration-dependent accumulation of acidic metabolites, bacteriocins, and other bioactive compounds present in the CFSs. Nevertheless, the higher IC_50_ values observed in CCD841 CoN cells compared to cancer cells further support the relative safety and selective cytotoxic activity of *Lactobacillus*-derived CFSs. While some studies have shown the cytotoxic effects of postbiotic metabolites at different incubation times, early cytotoxic effects have also been reported with short incubation periods, such as 24 h (Chuah et al., 2019; Nowak et al., 2022; Hoxha et al., 2023; Erfanian et al., 2023). In the present study, after 24 h of CFS application, the IC_50_ values calculated for healthy cells were significantly higher than those for cancer cell lines, indicating that both live and inactivated CFSs can exhibit a selective cytotoxic profile. In addition to cytotoxicity findings, AO/PI fluorescence staining analyses provided morphological confirmation of cellular death; fluorescence microscopy results showed increased apoptotic changes in colorectal cancer cell lines after CFS application, supporting the cytotoxicity data obtained from MTT analyses. However, the fact that inactivated CFS derived from *L. paracasei* exhibited stronger cytotoxic effects across all cell lines than the live form suggests that bacterial metabolites play a significant role in the emergence of this biological effect. In the current literature, no other studies have been found that examine the effects of CFS obtained from these two *Lactobacillus* strains on CCD841 CoN cells. This highlights the originality of the research in evaluating selective cytotoxicity and the safety profile.

In the literature, studies about the anticancer activity of CFSs on CRC cells using cell cycle analysis are limited (Chuah et al., 2019; Nowak et al., 2022; Erfanian et al., 2023; Hoxha et al., 2023; Salek et al., 2024). Dehghani et al. (2021) showed that *Lactobacillus rhamnosus*-induced CFS reduced viability in HT-29 cells in a time- and dose-dependent manner and caused cell cycle arrest, particularly in the G0/G1 and Sub-G1 phases. In another study, it was reported that CFS derived from *L. acidophilus* exhibited antiproliferative effects by arresting the cell cycle in the G1 phase and reducing the S and G2/M phases (Erfanian et al., 2023). Hu et al. (2015) reported that both live and inactivated *L. paracasei* M5L CFS suppressed growth in HT-29 cells, but the inactivated form showed a stronger anticancer effect by inducing apoptosis. Similarly, Huang et al. (2016) reported that CFSs increase apoptosis and suppress DNA replication and cell division by causing cell cycle arrest, particularly in the Sub-G and G0/G1 phases. The findings of the present study show that live and inactivated CFS derived from *L. bulgaricus* and *L. paracasei* significantly halted the cell cycle in the G0/G1 phase by reducing populations in the S and G2/M phases in HT-29 and Caco-2 cell lines. This suggests that these CFSs can effectively halt the cell cycle by suppressing cancer cell proliferation. Furthermore, the significant increase observed in the Sub-G1 population indicates apoptotic cell death. In contrast, the limited effect of these CFSs on the cell cycle of healthy CCD841 CoN cells suggests that these CFSs can selectively target colorectal cancer cells without harming healthy epithelial cells. All these results indicate that *Lactobacillus*-derived supernatants can be evaluated as a potential biotherapeutic agent by inducing apoptosis (Hu et al., 2015; Huang et al., 2016; Dehghani et al., 2021; Erfanian et al., 2023).

Research into genes involved in apoptosis plays a critical role in understanding the effects of probiotic bacteria on cancer cells (Karimi Ardestani et al., 2019; Haji Mehdi Nouri et al., 2023; Vachkova et al., 2023). Karimi Ardestani et al. (2019) reported that heat-inactivated *L. brevis* and *L. paracasei* strains induced apoptosis in HT-29 cells, with *L. brevis* showing a particularly strong effect. This effect has been shown to be associated with an increase in the Bax/Bcl-2 ratio, as well as Cas-3 and Cas-9 gene expression (Karimi Ardestani et al., 2019). Haji Mehdi Nouri et al. (2023) showed that *Lactobacillus casei-*derived CFSs suppressed proliferation in MCF-7 and HT-29 cells, and that this effect was associated with an increase in proapoptotic genes and a decrease in antiapoptotic genes at IC_50_ concentrations. Similarly, Vachkova et al. (2023) reported that *L. paracasei*-induced CFSs suppressed the growth of HT-29 cells and triggered apoptosis by altering the Bax/Bcl-2 ratio. The Bax/Bcl-2 ratio is a key indicator of the balance between proapoptotic and antiapoptotic signals within a cell, and an increase in this ratio is generally associated with the activation of mitochondrial pathway-mediated apoptosis. In the present study, it was observed that inactivated CFS obtained from *L. bulgaricus* and *L. paracasei* strains increased the expression of Bax, Cas-3, Cas-8, and Cas-9 genes and decreased the expression of the Bcl-2 gene in HT-29 and Caco-2 cells compared to live forms. These findings demonstrate that different *Lactobacillus* species can effectively suppress cancer cell growth and induce apoptosis. Therefore, these strains stand out as promising biotherapeutic agents that could potentially be used in the future. Furthermore, the ability of *Lactobacillus*-derived CFS to modulate the Bax/Bcl-2 balance is a possible mechanism underlying the antiproliferative and proapoptotic effects observed in colorectal cancer cells.

Despite these promising findings, it must be acknowledged that the study has some limitations. Specific bioactive compounds contributing to antitumor activity were not identified. Furthermore, the absence of a pharmacological positive control is another limitation, potentially restricting the comparative interpretation of the findings. Additionally, cellular responses were assessed at a single 24-h time point to detect early-phase effects; time-course analyses across multiple time points could not be performed.

In conclusion, the findings of the present study suggest that probiotic-derived CFSs may be a promising option for cancer treatment due to their selective effects on cancer cells and low toxicity profile in healthy cells. Moreover, in light of these results, it is thought that CFSs derived from probiotic bacteria could offer an innovative approach to conventional cancer treatments.

## Figures and Tables

**Table:** Primer sequences and NCBI reference numbers for genes.

Genes	Forward primers (5′->3′)	Reverse primers (5′->3′)	NCBI number
BAX	GGAGCTGCAGAGGATGATTG	GGCCTTGAGCACCAGTTT	NM_001291429.2
Cas-9	CGACCTGACTGCCAAGAAA	CATCCATCTGTGCCGTAGAC	NM_001229.5
Cas-8	GCCCAAACTTCACAGCATTAG	GTGGTCCATGAGTTGGTAGATT	NM_001080124.2
Cas-3	GAGCCATGGTGAAGAAGGAATA	TCAATGCCACAGTCCAGTTC	NM_001354779.2
Bcl-2	GTGGATGACTGAGTACCTGAAC	GAGACAGCCAGGAGAAATCAA	NM_000633.3
GAPDH	TGAACGGGAAGCTCACTGG	TCCACCACCCTGTTGCTGTA	NM_001256799.3
